# Supplementary material for: Resolving the full spectrum of human genome variation using Linked-Reads
Source: Genome Res. 2019 Apr;29(4):635–45. doi: 10.1101/gr.234443.118 (PMC6442396; doi:10.1101/gr.234443.118)
Supplement: Supplemental Material [file supp_gr.234443.118_Supplemental_longranger-2.2.zip › longranger-2.2.tar.bz2!/lr2.2-public/lib/stan/cmdstan/stan/doc/api/html/stored__gradient__vari_8hpp_source.html]

Stan: src/stan/agrad/rev/matrix/stored\_gradient\_vari.hpp Source File


|  |  |
| --- | --- |
|  | Stan  2.5.0  probability, sampling & optimization |


- Main Page
- Related Pages
- Namespaces
- Classes
- Files

- File List
- File Members

All Classes Namespaces Files Functions Variables Typedefs Enumerations Enumerator Friends Macros Pages

- src
- stan
- agrad
- rev
- matrix

stored\_gradient\_vari.hpp

Go to the documentation of this file.

1 #ifndef STAN\_\_AGRAD\_\_REV\_\_MATRIX\_\_STORED\_GRADIENT\_HPP

2 #define STAN\_\_AGRAD\_\_REV\_\_MATRIX\_\_STORED\_GRADIENT\_HPP

3

4 #include <stan/agrad/rev/vari.hpp>

5

6 namespace stan {

7

8  namespace agrad {

9

18  class stored\_gradient\_vari : public vari {

19

20  protected:

21

22  size\_t size\_;

23  vari\*\* dtrs\_;

24  double\* partials\_;

25

26  public:

27

38  stored\_gradient\_vari(double value,

39  size\_t size,

40  vari\*\* dtrs,

41  double\* partials)

42  : vari(value),

43  size\_(size),

44  dtrs\_(dtrs),

45  partials\_(partials) {

46  }

47

52  void chain() {

53  for (size\_t i = 0; i < size\_; ++i)

54  dtrs\_[i]->adj\_ += adj\_ \* partials\_[i];

55  }

56  };

57

58  }

59 }

60

61 #endif

stan::agrad::stored\_gradient\_vari::partials\_

double \* partials\_

**Definition:** stored\_gradient\_vari.hpp:24

vari.hpp

stan::agrad::stored\_gradient\_vari::chain

void chain()

Propagate derivatives through this vari with partial derivatives given for the daughter vari by the s...

**Definition:** stored\_gradient\_vari.hpp:52

stan::agrad::stored\_gradient\_vari::dtrs\_

vari \*\* dtrs\_

**Definition:** stored\_gradient\_vari.hpp:23

stan::agrad::stored\_gradient\_vari

A var implementation that stores the daughter variable implementation pointers and the partial deriva...

**Definition:** stored\_gradient\_vari.hpp:18

stan::agrad::stored\_gradient\_vari::size\_

size\_t size\_

**Definition:** stored\_gradient\_vari.hpp:22

stan::agrad::vari

The variable implementation base class.

**Definition:** vari.hpp:28

stan::math::size

int size(const std::vector< T > &x)

**Definition:** size.hpp:11

stan::agrad::stored\_gradient\_vari::stored\_gradient\_vari

stored\_gradient\_vari(double value, size\_t size, vari \*\*dtrs, double \*partials)

Construct a stored gradient vari with the specified value, size, daughter varis, and partial derivati...

**Definition:** stored\_gradient\_vari.hpp:38

stan::agrad::vari::adj\_

double adj\_

The adjoint of this variable, which is the partial derivative of this variable with respect to the ro...

**Definition:** vari.hpp:43


---

[ Stan Home Page ]

*© 2011–2014,
Stan Development Team.*
